# The simulation and experimental validation of a novel noninvasive multi-target electrical stimulation method

**DOI:** 10.1038/s41598-024-61571-9

**Published:** 2025-04-11

**Authors:** Kai Zhu, Xiaoqing Zhou, Xu Liu, Ren Ma, Mingpeng Wang, Shunqi Zhang, Tao Yin, Zhipeng Liu

**Affiliations:** 1 Institute of Biomedical Engineering, Chinese Academy of Medical Sciences & Peking Union Medical College, Tianjin, China; 2 Tianjin Polytechnic University, Tianjin, China

**Keywords:** Noninvasive neuromodulation, Multi-target, Electrical stimulation, High spatial resolution, Neuroscience, Biomedical engineering, Neurological disorders

## Abstract

The brain is a complex system of structure and function. Brain diseases and brain functional abnormalities often involve multiple functionally connected regions, including the deep brain. Studies have shown that multi-target electrical stimulation is more effective than single-target electrical stimulation. However, non-invasive multi-target electromagnetic stimulation, such as multi-target transcranial magnetic stimulation (TMS), transcranial direct current stimulation (tDCS) and transcranial alternating current stimulation (tACS) cannot meet the needs of synchronous multi-target accurate electrical stimulation at the deep brain. In this paper, based on the principle of magneto-acoustic coupling and phased array focusing technology, a novel non-invasive multi-target transcranial magneto-acoustic coupling electrical stimulation (multi-target TMAES) method is proposed. A simulation model and experimental system were established. The simulation and experimental results proved that the proposed multi-target TMAES can non-invasively achieve precise focused electrical stimulation of two targets. The average focal point size of each target is 5.1 mm. The location and intensity of the multi-target electrical stimulation can be flexibly changed by adjusting the system parameters according to the actual need. It will provide a new and promising tool for the treatment of brain diseases and the study of neural circuits and brain functional connectivity.

## Introduction

As a structurally and functionally complex system^1^, the brain usually requires multiple regions and nuclei to work in concert when performing tasks, and functional abnormalities usually involve multiple functionally connected regions^2,3^. Usually, there is a functional connection between the deep area and multiple superficial cortex^4^. It is widely believed that the occurrence of functional brain diseases is often associated with neuronal loss, abnormal discharges and neural circuit disorders in multiple locations of the brain^5^. Therefore, simultaneous multi-target precision neuromodulation of multiple brain regions can achieve more positive results than single-target stimulation.

Karlo et al. show that bilateral subthalamic nucleus deep brain stimulation improves motor and gait scores substantially more than unilateral^6^. Weise et al. found that symptomatic hyperkinetic movement disorders can be improved by multi-target deep brain stimulation of subthalamic nucleus (STN) and medial globus pallidus nucleus (GPI)^7^. However, DBS is an invasive technique. In the more concerned area of non-invasive neuromodulation, Lehner et al. studied that the effect of multi-target TMS on auditory cortex is better than that of single target stimulation^8^ in treatment of tinnitus disease. In addition, Kleinjung et al. found using multi-target transcranial magnetic stimulation (TMS) can produce a longer-term therapeutic effect than the standard temporal lobe stimulation regime in the regulation of prefrontal and temporal cortex activity^9^. Furthermore, in 2021, Zhang et al.^10^ used multi-target transcranial direct current stimulation (tDCS) to stimulate prefrontal areas, the left dorsolateral prefrontal cortex (DLPFC) and bilateral frontotemporoparietal cortex (FTPCs) in patients with long-term disorders of consciousness (DOC) and examined alterations in cortical interconnections using nonlinear electroencephalography (EEG). Saturnino et al.^11^ used dual-site transcranial alternating current stimulation to modulate synchronisation between theta-band frontal pole and parietal cortical regions, thereby altering visual memory matching activity. Miyaguchi et al.^12^ used transcranial alternating current stimulation (tACS) with gamma bands to stimulate both the M1 and cerebellar hemispheres more efficiently than stimulating each region individually to improve motor function. Due to the equipment and focus limitations, the above non-invasive multi-target technology (TMS, tDCS and tACS) often employs non-synchronous stimulation^9–12^.

Although in 2022, Memarian et al.^13^ proposed and designed a dual-channel modulation-based magnetic pulse generator and a novel coil arrangement that enables simultaneous multi-target TMS. Nevertheless, focusing of electric field resolution on the order of mm is difficult to achieve by TMS, and focusing is inversely proportional to the depth of stimulation, making it difficult to achieve direct stimulation of the deeper parts of the brain. TDCS and tACS are inferior to TMS in terms of the focusing performance and stimulation depth. Therefore, when these electrical stimulation techniques are applied to multi-target electrical stimulation, the impact of brain overlap will be more serious, and it is more difficult to directly regulate deep brain regions^11,14–16^. Moreover, the targets of the above multi-target TMS, tDCS and tACS are all in the cortex. And these techniques all suffer from technical bottlenecks in terms of focusing and depth of stimulation^17^. TIS is a promising technique for the treatment of neuromotor disorders, with superior focality, steerability, and tolerability compared to traditional electrical stimulation^18^. However, current human trials have produced fewer and inconsistent results, so animal and simulated experiments are still needed to refine the stimulation regimen for human trials. Second, most current studies are based on the citation analysis, method application and prospect of TIS stimulation patterns and conclusions proposed by Grossman, and have not yet supplemented and improved the existing problem of insufficient focus. In addition, the essence of TIS is still transcranial electrical stimulation technology, and it still has the original defect of the current diffusion in the brain in the transcranial electrical stimulation technology^19,20^.

In recent years, transcranial ultrasound stimulation (TUS) has attracted extensive interest due to its advantages of high stimulation resolution and high stimulation depth^18^. Currently, there are also a few studies that apply the TUS to achieve multi-target stimulation. Zhuang et al.^19^ developed a high-power ultrasound excitation system in 2021 that can generate multiple ultrasound-focused targets at the same time and carried out ex vivo acoustic field testing using a rat skull. He et al.^20,21^ demonstrated in vivo that the microunit-piece transducer and micro acoustic holographic lens ultrasound neuromodulation system can achieve dual-target ultrasound neuromodulation in awake small animals. However, ultrasound stimulation is a type of mechanical modulation^22,23^. Most of the existing studies on the effect of multi-target stimulation are based on electromagnetic stimulation. Compared with the electromagnetic modulation methods based on neurophysiology, the mechanism of mechanical stimulation is still in the process of exploration. At present, the effectiveness and benefits of multi-target ultrasound stimulation in the treatment of different brain diseases still need to be evaluated. The dual-target or even multi-target TUS cannot be directly applied to the more mature electromagnetic stimulation network nodes. Therefore, the simultaneous implementation of multi-target electrical stimulation techniques to directly stimulate deep brain regions is still very challenging.

Transcranial magneto-acoustic stimulation (TMAS) is a novel multiphysics coupling neuromodulation technique that was first proposed by Norton^24^ in 2003, which using the principle of magneto-acoustic coupling to combine an ultrasound field and a static magnetic field for noninvasive neuroelectric stimulation. Until the beginning of 2016, the effectiveness of TMAS has been continuously proven to improve memory and synaptic plasticity, improve motor ability in mice, and could play an important role in the treatment. Wang et al.^25^ demonstrated that TMAS can shorten the response time of neural activity and enhance the neuromodulator effect of TUS on the motor cortex. Chu et al. ^22^ demonstrated that TMAS treatment enhanced autophagy through the activation of microglial Piezo1 cells, promoting phagocytosis and degradation of β-amyloid and alleviating neuroinflammation, synaptic plasticity damage and neural oscillatory abnormalities in 5xFAD mice, showing stronger effects than TUS.

In this paper, aiming at the needs of neuromodulation and solving the shortcomings of existing multi-target stimulation technology, a new noninvasive multi-target transcranial magneto-acoustic coupling electrical stimulation (TMAES) method is proposed, based on the principle of magneto-acoustic coupling effect and phased array focusing technology, which can realize a number of independent and high spatially resolution focused electrical stimulations at the deep brain. We established a multi-target TMAES stimulation model and experimental system. The multiple focused electric field distribution and intensity are detected, calculated and analyzed. The proposed multi-target TMAES method can achieve multiple targets (≥ 2) of high-spatial resolution (mm-level) electrical stimulation at deep brain (50 mm). Moreover, based on the simulated and experimental data, we also probed that the decrease of stimulation energy in multi-target focusing can be compensated by increasing magnetic induction intensity rather than only increasing the ultrasonic excitation intensity. It will provide a prominent tool for exploring the brain functional connectivity in the pathogenesis of major encephalopathy and rehabilitation treatment.

## Methods

### Principle of magnetic-acoustic coupling effect

**Figure 1:**
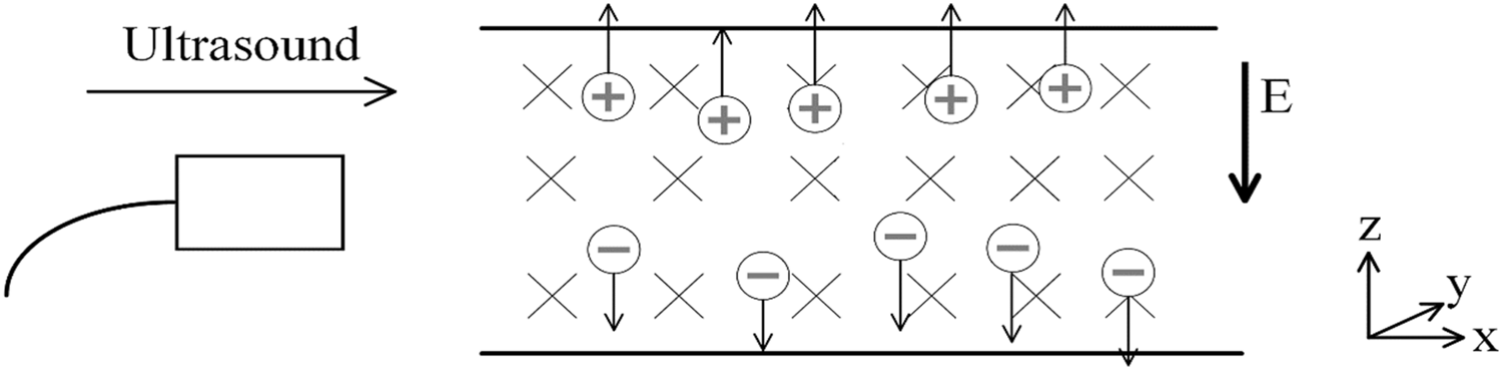
The schematic of the magneto-acoustic coupling effect.

Multi-target TMAES is based on the magneto-acoustic coupling effect, using the high focusing performance of ultrasound under the condition of a loading magnetic field to achieve high spatial resolution noninvasive electrical stimulation^26^. Conductive particles in biological tissue vibrate under ultrasonic excitation. In the case of the existence of a magnetic field (usually a steady magnetic field), perpendicular to the vibration direction of the particles the conductive particles are affected by the Lorentz force, and the positive and negative particles deflect and separate at the focus area along the direction of vector product of the steady magnetic field and the ultrasonic field, forming an internal induced electric field, magneto-acoustic electric field (MAEF)^27^. The schematic of magneto-acoustic coupling effect is shown in Fig. 1.

The propagation of ultrasound satisfies the acoustic pressure wave equation,

1 $$ \nabla^{2} p({\varvec{r}},t) - \frac{1}{{c_{s}^{2} }}\frac{{\partial^{2} p({\varvec{r}},t)}}{{\partial t^{2} }} = S({\varvec{r}},t) $$

where $S(r,t)$ is the acoustic source and p is the acoustic pressure.

The longitudinal pressure wave propagating along the x-axis follows the fluctuation equation

2 $$ \frac{{\partial^{2} x}}{{\partial z^{2} }} = \frac{1}{{c_{0}^{2} }}\frac{{\partial^{2} x}}{{\partial t^{2} }} $$

where *x* is the distance of the particle from its equilibrium position and $c_{0}$ is the ultrasonic propagation velocity.

In the case of a forward sinusoidal wave, the instantaneous velocity of the particle along the x-axis is $v_{x}$,

3 $$ v_{x} = \frac{\partial x}{{\partial t}} = V\sin (wt - \varphi ) $$

where $V$ is the magnitude of the particle vibration velocity, $w = 2\pi f$ is the angular frequency, and $f$ is the ultrasonic frequency, and $\varphi$ represents the phase angle.

The relationship between the acoustic pressure *P* of the ultrasonic wave propagating into the tissue and the vibrational velocity of the particle in the tissue can be expressed as

4 $$ P = \rho c_{0} v_{x} $$

where $\rho$ is the tissue density and $c_{0}$ is the propagation speed of ultrasound in tissue.

According to the theory of Montalibert and Norton^24,28^, the current density $J$ along the y-axis generated by ultrasound and a static magnetic field in biological media can be derived and expressed as

5 $$ J_{{}} = \sigma v_{{\varvec{x}}} B_{y} $$

6 $$ J = q(n^{ + } u^{ + } + n^{{_{ - } }} u^{ - } )v_{x} B_{y} $$

7 $$ \sigma = q(n^{ + } u^{ + } + n^{ - } u^{ - } ) $$

where σ is the conductivity of the tissue, *n*^+^ and *n*^-^ are the concentrations of the positive and negative ions, *u*^+^ and *u*^-^ are the mobilities, respectively, and $B_{y}$ is the magnetic field.

In the presence of a steady magnetic field $B_{y}$, the internal electric field force $F_{1} = qE_{Z}$ and the Lorentz force $F_{2} = qv_{x} B_{y}$ are in equilibrium, which can be obtained as

8 $$ E_{z} = v_{x} B_{y} $$

where q is the electric charge. The electric field intensity can be obtained as

9 $$ E_{z} = \frac{1}{{\rho c_{0} }}P_{x} B_{y} $$

### Focusing law of the phased array

**Figure 2:**
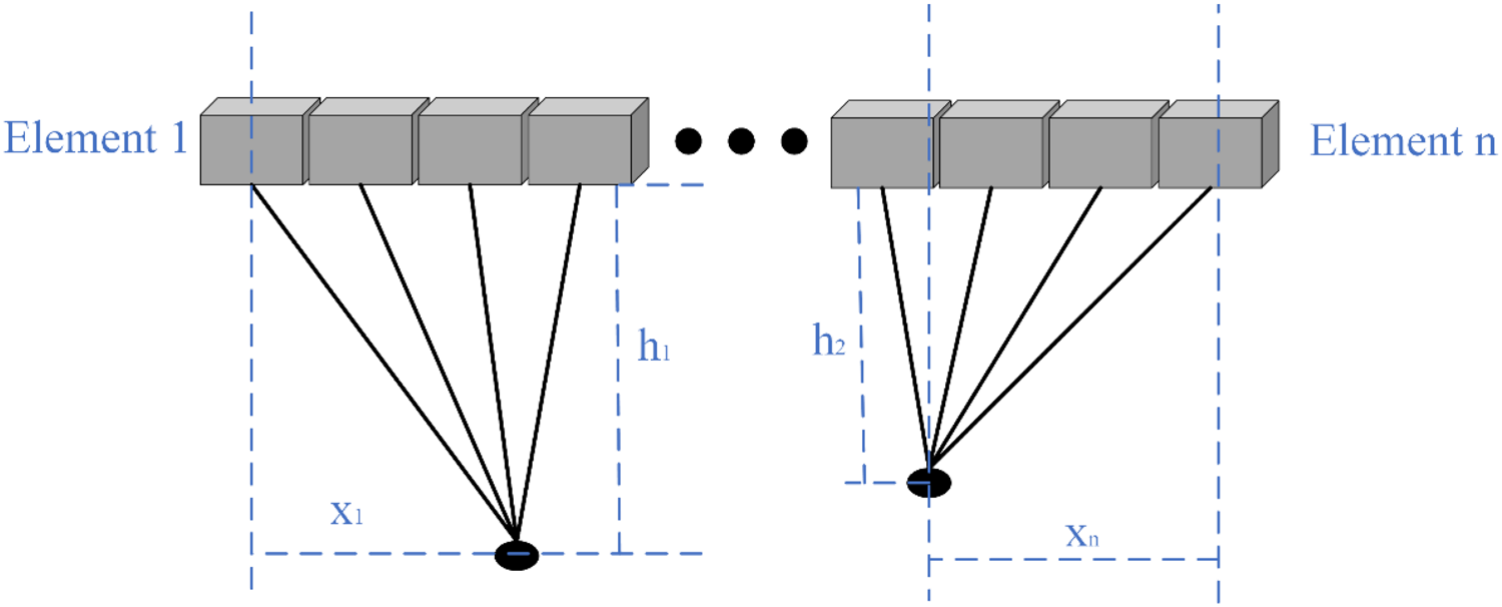
Schematic diagram of phased array in dual-target focusing.

TMAES uses an ultrasound field and a static magnetic field to generate an induced electric field, and therefore changes the distribution of the induced electric field by controlling the focusing effect of the ultrasound. Commonly, phased-array focusing is achieved by adjusting the delay of each element to deflect and focus the ultrasound^29^. Multi-target focusing needs to consider the structure and size of the phased array transducer, and set the positional coordinates of each focusing point then calculate the delay of each element is calculated according to Eqs. (8) and (9), respectively. Taking the dual-target focusing point as an example, a schematic diagram is shown in Fig. 2.

10 $$ t_{i1} = \frac{{\sqrt {x_{i1}^{2} + h_{i1}^{2} } }}{{c_{0} }} $$

11 $$ t_{j2} = \frac{{\sqrt {x_{j2}^{2} + h_{j2}^{2} } }}{{c_{0} }} $$

In Eqs. (10) and (11), $c_{0}$ is the ultrasound propagation velocity,$x_{i1}$ is the horizontal distance between focusing point 1 and the corresponding i-th element centre, $h_{i1}$ is the vertical distance between focusing point 1 and the i-th element center, $x_{j2}$ and $h_{j2}$ is the horizontal distance and vertical distance between the focusing point 2 and the *j*-th element center, respectively. And* t*_i1_ denotes the focusing time of the array element at focusing point 1.* t*_j2_ denotes the focusing time of the array element at focusing point 2.

### Establishment of the simulation model

**Figure 3:**
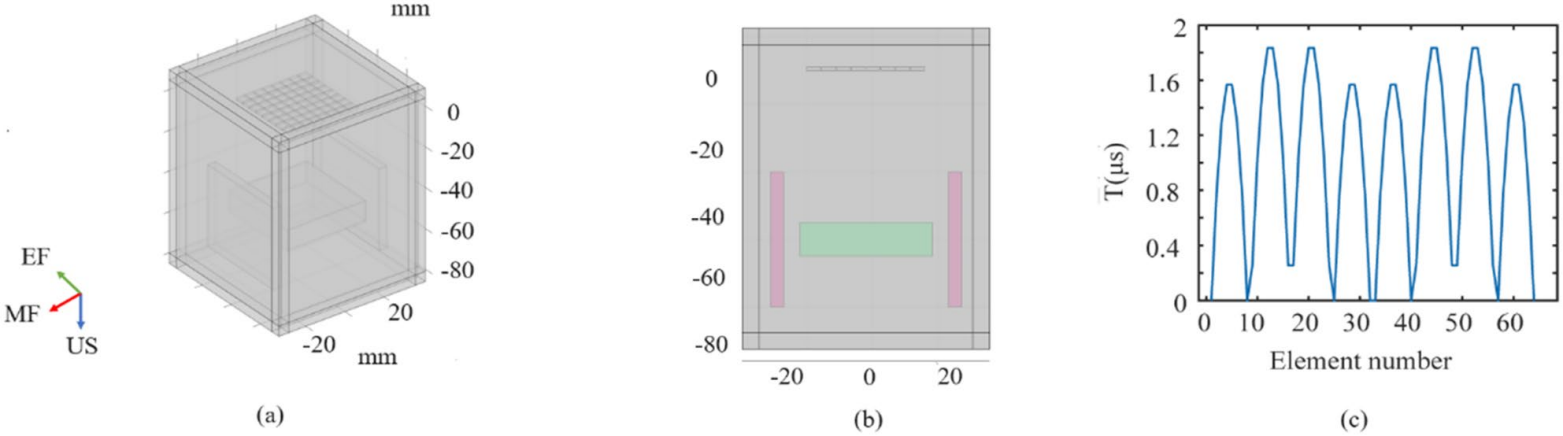
(**a**) Simulation model diagram. (**b**) Longitudinal cross-section of the model. (**c**)Time delay of the 64 elements in array transducer.

The pressure acoustics, magnetic field, and electric field modules in COMSOL were used to establish a multi-target TMAES model^30^, which is shown in Fig. 3a,b. The top is the phased array transducer, the bottom pink area is the static magnet, and the green area is the water that we set up to simulate the biological tissue. The model consists of a 64 elements plane array transducer, a device that generates a magnetic field, and a tissue like water body. Among them, the structure and frequency parameters are shown in Table 1, and the magnet device is composed of two 45 mm × 45 mm × 5 mm rectangular models, which can generate a uniform magnetic field of 0.5 T in the central area, and the water body area is 75 mm × 75 mm × 85 mm. The electrical conductivity of the water body is set to be 0.5 S/m, which is the same as that of the neural tissues, and the acoustic velocity is 1500 m/s. The maximum cell size is 2.63 mm and the minimum cell size is 0.113 mm for the dissected mesh. According to the formula, based on the two focusing spatial coordinates of the target point, the element with the longest distance from the acoustic wave propagation to the focal point is selected as the reference element. The time delay of each element is calculated relative to it to obtain the emission delay of the 64 elements. The time delay diagram is shown in Fig. 3b.

**Table 1:** Parameters of the 64-element plane array transducer.

Parameters	
Horizontal axis element center distance	4.5 mm
Longitudinal axis element center distance	4.5 mm
Element spacing	0.2 mm
Frequency	0.5 MHz
Structure of array elements	8 × 8

### Establishment of the experimental system

**Figure 4:**
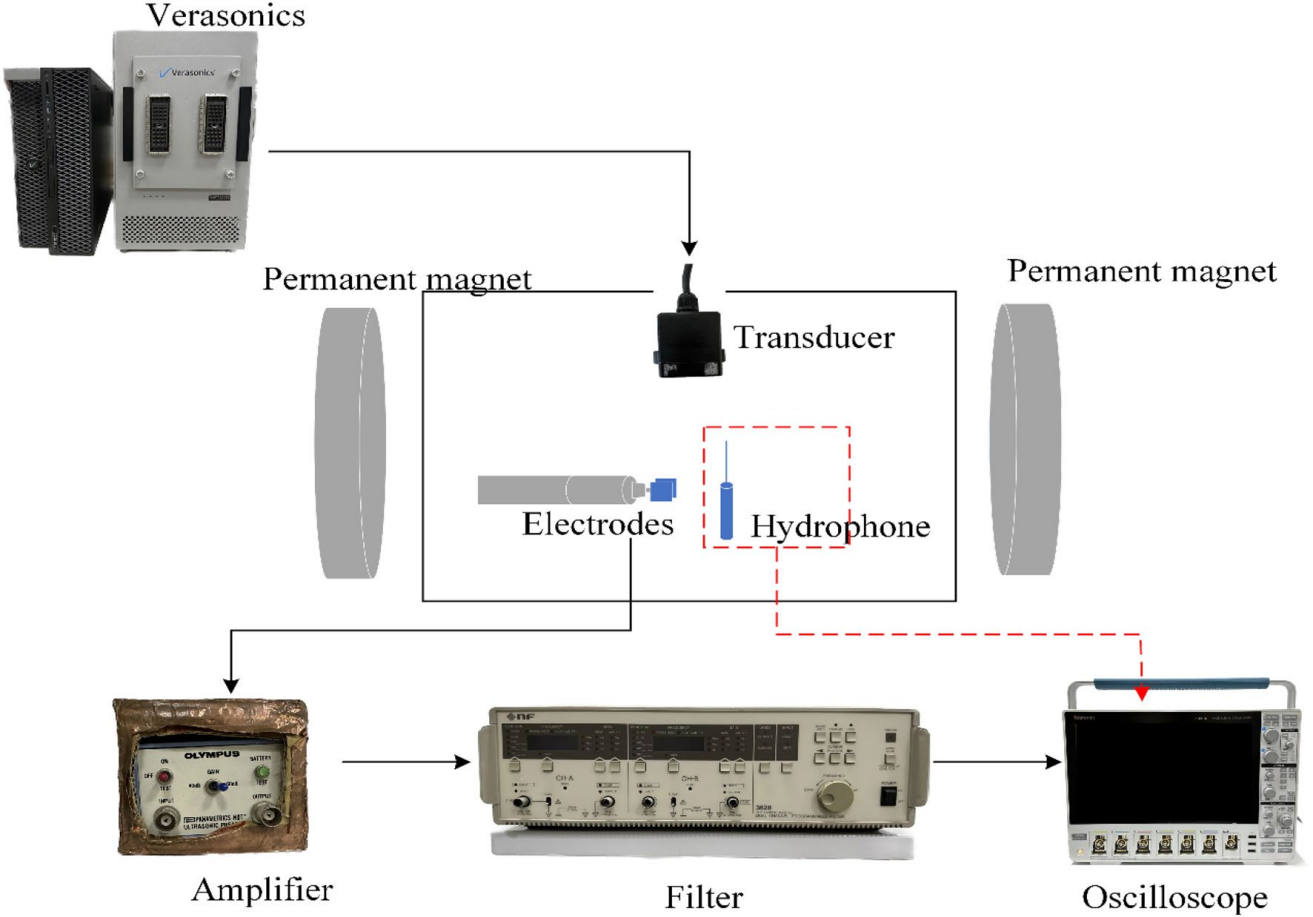
(**a**) Schematic of the multi-target TMAES measurement system. (**b**) Picture of the self-made electrode.

To validate the stimulation results of multi-target TMAES, we build a multi-target acoustic-electrical detection system, which can detect the distribution of the acoustic pressure and electric field intensity under different focusing targets. As shown in Fig. 4a, a Verasonics Vantage system (Verasonics 256, Verasonics Inc., USA) controls an array transducer (HP-500k-64, Doppler, China) to emit 500 kHz single pulse ultrasound, a hydrophone (NH1000, Precision acoustic, U.K.) is placed 50 mm below the transducer to detect acoustic pressure inside the water tank. The experimental ultrasonic transducer dimensions and other details are consistent with those used in the simulation. We used a 2D surface phased array ultrasound transducer in our experiments. It has a square surface and consists of 64 arrays of square elements arranged in an 8 × 8 manner. In addition, a static magnet was placed on each of the two opposite sides of the water tank to generate a uniform magnetic field of 0.5 T. The vibrations of the charged particles triggered by the ultrasound can generate an induced electric field under the magnetic field, which can be detected by a self-made silver plate electrodes (shown in Fig. 4b). The size of each single plate of the electrodes is 10 mm × 1 mm × 0.1 mm. The distance between the two plates is 1 mm. Because the voltage signals of the magneto-acoustic electrical stimulation detected by the electrodes are very weak and noisy, they need to be amplified by using an amplifier (5660C, Olympus, Japan) with 60 dB gain and then filtered by a filter (3628, NF, Japan), with the bandpass band of 400–600 kHz. The amplified and filtered electrical signals are sampled averaged 200 times on an oscilloscope (MSO46, Tektronix, America). The voltage signals were collected and displayed to obtain the electric field intensity.

## Results

### Simulation results

**Figure 5:**
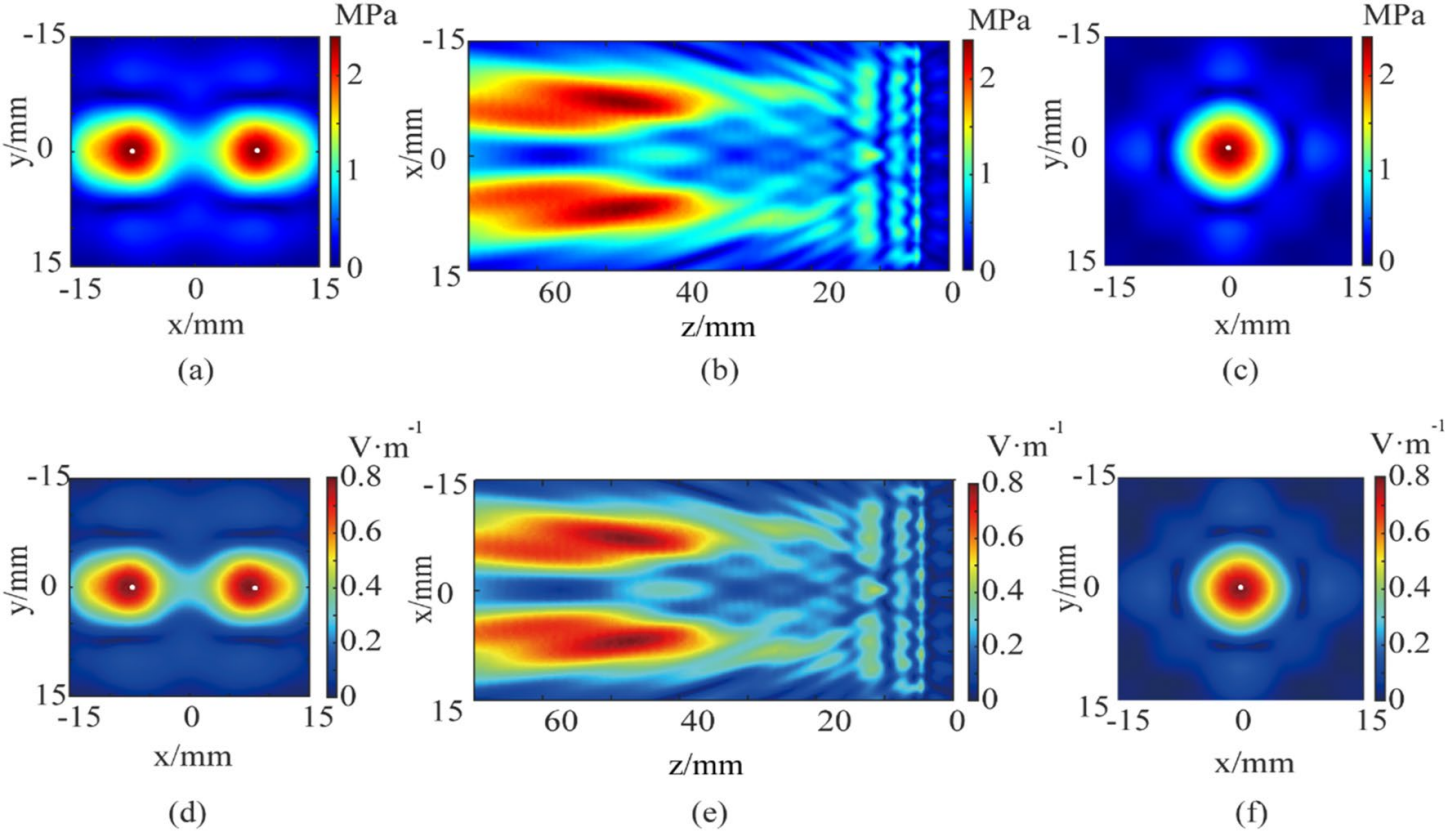
(**a**) Dual-target xy-plane acoustic pressure field distribution at z = 50 mm; (**b**) xz-plane acoustic pressure field distribution at y = 0 mm; (**c**) single-target xy-plane acoustic pressure field distribution at z = 50 mm; (**d**) dual-target xy-plane electric field intensity distribution at z = 50 mm; (**e**) xz-plane electric field intensity distribution at y = 0 mm; (**f**) single-target xy-plane electric field intensity distribution at z = 50 mm. The white dot is the set focus point.

Using the simulation model in Fig. 2, the ultrasonic waves emitted by the ultrasonic transducer are focused through the water at the two preset focal points away from the transducer surface 50 mm. According to the transducer array parameters and distribution symmetry, the focus coordinates are set to (− 9, 0, − 50) and (9, 0, − 50) respectively. The ultrasound is simulated by using the pressure acoustics module, and the magneto-acoustic coupling electric field is simulated with the magnetic field and electric field module. The main frequency of the ultrasound is 500 kHz, and the magnetic induction intensity is 0.5 T. The dual-target acoustic pressure distribution in the xy-plane at z = 50 mm and in the xz-plane at y = 0 mm are shown in Fig. 5a,b. The dual-target electric field intensity distribution in the xy-plane at z = 50 mm and in the xz-plane at y = 0 mm are shown in Fig. 5d,e. In addition, the single-target magneto-acoustic electrical stimulation is simulated and compared under the same conditions, the acoustic field distribution and electric field distribution are as shown in Fig. 5c,f, with the focal point coordinates of (0, 0, − 50). The region formed by the peak-to-peak attenuation of 3 dB of acoustic pressure and electric field intensity is defined as the focus area. Figure 5a,d, it is calculated that the dual-target total focusing area of the acoustic pressure field is 65.0 mm^2^ and the total focusing area of the electric field is 65.0 mm^2^. It can be seen that the acoustic pressure and electric field intensity at the two targets are symmetrically distributed, which is consistent with the transducer focusing setting. It is calculated that the acoustic field focusing area of each target is 32.5 mm^2^, and the electric field focusing area is also 32.5 mm^2^. The maximum acoustic pressure is 2.5 MPa and the maximum electric field intensity is 0.8 V/m for dual-target stimulation. According to Fig. 5c,f, the single-target focusing area of acoustic pressure field and electric field are both 33.7 mm^2^. Then, the maximum acoustic pressure of single-target is 2.7 MPa, and the maximum electric field intensity is 0.9 V/m. In Fig. 5a, the focusing length of the long axis of the left focal point in the xy plane is 7.1 mm and that of the short axis is 4.3 mm. The dual-target total focusing area of the acoustic pressure field is 65.0 mm^2^. The focus length of the long axis on the right is 7.0 mm, and the focus length of the short axis is 4.4 mm. In Fig. 5d, in the xy plane, the focus length of the left focus axis is 7.1 mm and the focus length of the short axis is 4.3 mm. The focus length of the long axis on the right is 7.0 mm, and the focus length of the short axis is 4.4 mm.

The data show that whether it is single-target or dual-target stimulation, the distribution of acoustic pressure field and electric field intensity are consistent. Compared with the single-target stimulation under the same excitation conditions and the same array transducer, the acoustic pressure and electric field intensity of the dual-target stimulation are decreased, but the spatial resolution of each target, that is, the size of each target, has improved. As shown in Fig. 5d, the method can form two effective electrical stimulation targets at the set positions of (− 9, 0, − 50) and (9, 0, − 50), which shows the dual-target TMAES can achieve mm-level accurate focusing at the deep brain. Moreover, based on the symmetrical focusing algorithm, the shape, area, and energy of the two stimulus targets are similar.

**Figure 6:**
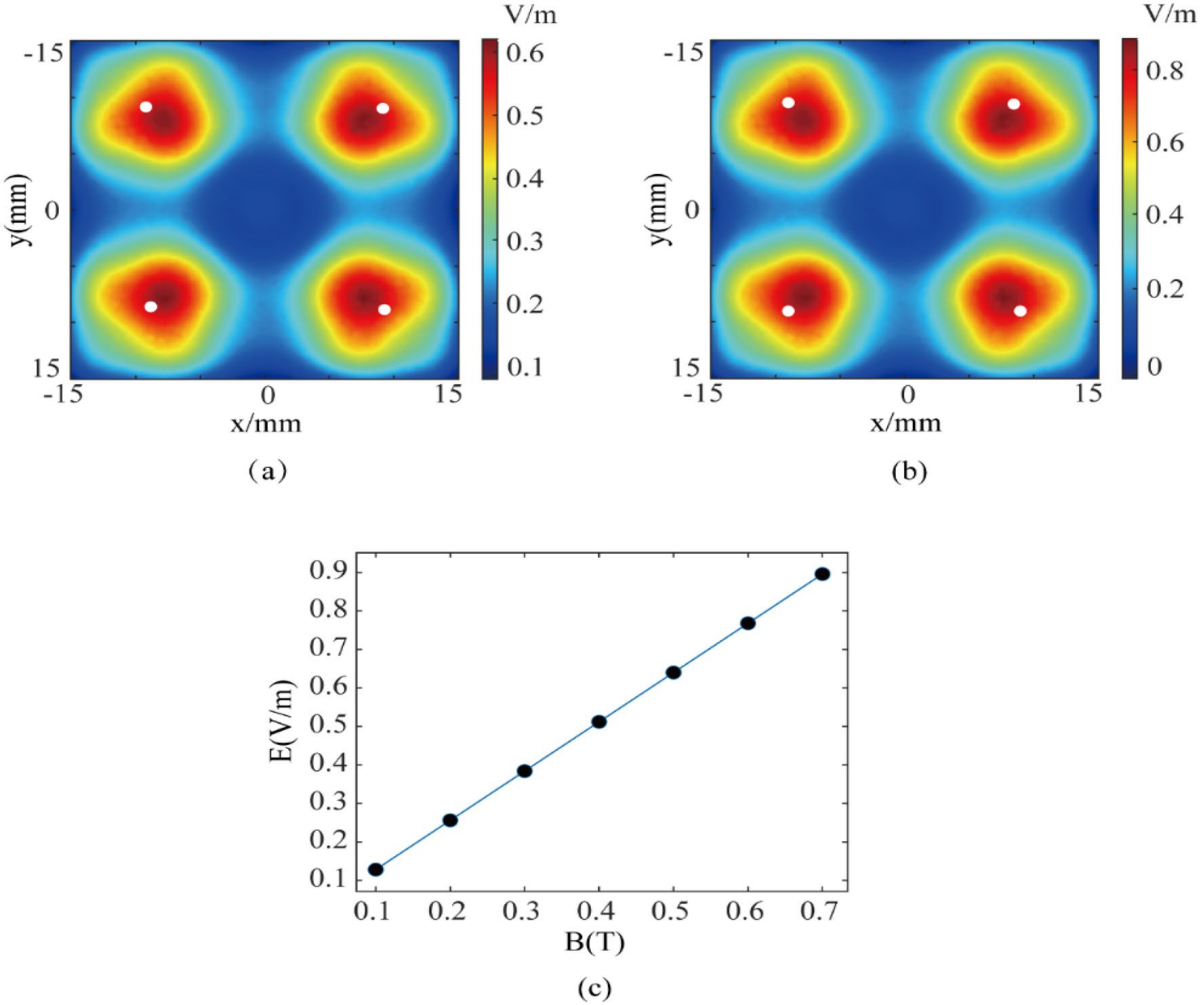
Distribution of the stimulated electric field intensity at quad-target when (**a**) B = 0.5 T and (**b**) B = 0.7 T. The white dot is the set focus point. (**c**) The intensity of magneto-acoustic coupled electric field at different magnetic induction intensities.

To demonstrate the feasibility of electrical stimulation with more focusing targets, based on the specification of the 64-element phased-array transducer, we further establish quad-target magneto-acoustic electrical stimulation model and perform corresponding simulations. The focal coordinates are set as (− 9, 9, − 50), (9, 9, − 50), (9, − 9, − 50), and (− 9, − 9, 50), the main frequency of the ultrasound is 500 kHz, and the magnetic induction intensity is 0.5 T. The quad-target simulation result of electric field intensity distribution is shown in Fig. 6a. The data show that four effective electrical stimulation targets are indeed produced at the four focal points set. The total focal area is 168.1 mm^2^. The focal area of the single target area is 42.0 mm^2^, which is approximately 1/4 of the total area. The maximum electric field intensity is 0.64 V/m. In Fig. 6a, the average long-axis focusing length of each target was 4.4 mm and the short-axis focusing length was 5.3 mm. In Fig. 6b, the average long-axis focusing length of each target was 4.4 mm and the short-axis focusing length was 5.3 mm.

Compared with dual-target stimulation and single-target stimulation, Fig. 6a shows that the focal area of each target in the quad-target stimulation has increased. It is because as the increase of the number of focusing targets, the number of arrays corresponding to each target involved in focusing decreases. Therefore, the effect of the number of focusing elements on the focusing region should be considered when performing multi-target design. In addition, the maximum electric field intensity also gradually decreases as the number of targets increases, because the ultrasonic energy involved in focusing decreases. However, based on the law of focusing, the change of electric field intensity in single-target, dual-target and quad-target modes does not scale linearly with the number of excitation elements.

In multi-target TUS, the problem of stimulation intensity weakening of single target can be overcome by increasing acoustic pressure or frequency. While in multi-target TMAES, according to Eq. (9), the electric field intensity can be compensated by increasing the magnetic induction intensity, so as to achieve the ideal electrical stimulation intensity. When the main frequency of ultrasound is 500 kHz, by changing the magnetic induction intensity (B = 0.7 T) of the focal region, the maximum electric field intensity is 0.9 V/m. The simulation results are shown in Fig. 6b. We can see that the magneto-acoustic coupling electric field intensity is proportional to the magnetic induction electric field intensity in Fig. 6c. From Eq. (9), we can derive that E and B are always positively related, and therefore the plot ***E*** vs ***B*** applies to all multi-target TMAES techniques. Therefore, in multi-target TMAES, each target of multi-target stimulation can reach the electric field intensity of single target stimulation without increasing the ultrasonic intensity, that is, there is no loss of electric field intensity.

**Figure 7:**
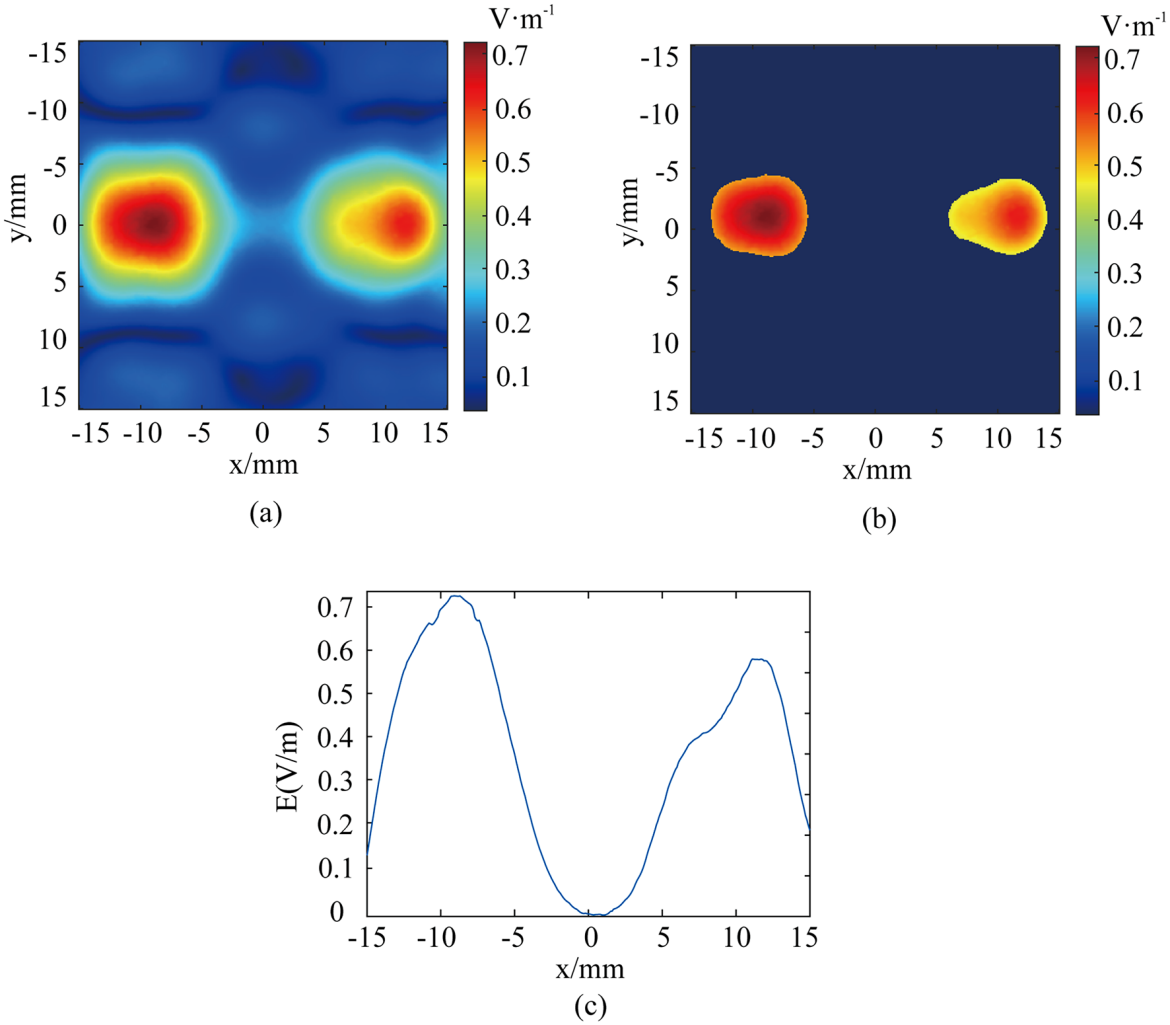
Distribution of the (**a**) xy-plane electric field intensity and (**b**) electric field focusing region (− 3 dB) for different target stimulus intensities and (**c**) x-axis line electric field intensity distribution. The white dot is the set focus point.

The above simulations are all based on the case of the targets with symmetric distribution. However, there are systematic differences in threshold stimulation intensities corresponding to different regions of the human brain. Considering the possible variability of the electrical stimulation intensity that is required for different brain regions or nuclei in practical applications, the dual-target electrical stimulation scheme above is improved. According to the specification parameters of the transducer array elements and the principle of focusing, the focal coordinates are set to (− 9, 0, − 50) and (− 13.5, 0, − 50), respectively. The main frequency of the ultrasound is 500 kHz and the magnetic induction is 0.5 T. There are 32 elements participating in focusing on the left target point, and 16 elements participating in focusing on the right target point. The electric field intensity distribution in the xy-plane is shown in Fig. 7a. Based on the definition of the focusing region above, the distribution of the focusing region is plotted as shown in Fig. 7b. The corresponding x-axis distribution of the electric field intensity at y = 0 is drawn, and Fig. 7c is obtained. As shown in Fig. 7, the distribution of electric field intensity at the two focal points is clearly different. The long axis focusing length of the left target is 9.2 mm and the short axis focusing length is 6.2 mm. The long axis focusing length of the right target is 8.6 mm, and the short axis focusing length is 4.8 mm. The maximum electric field intensity at the left focus is 0.72 V/m and the focus area is 43.4 mm^2^. The maximum electric field intensity at the right focus is 0.62 V/m and the focus area is 37.6 mm^2^. The positions of the maximum electric field intensity at the left and right focuses are 20 mm apart. This shows that multi-target TMAES can achieve different intensities electrical stimulation at different targets by changing the ultrasound focusing scheme with an appropriate focusing effect while maintaining mm-level resolution.

### Experimental results and analysis

**Figure 8:**
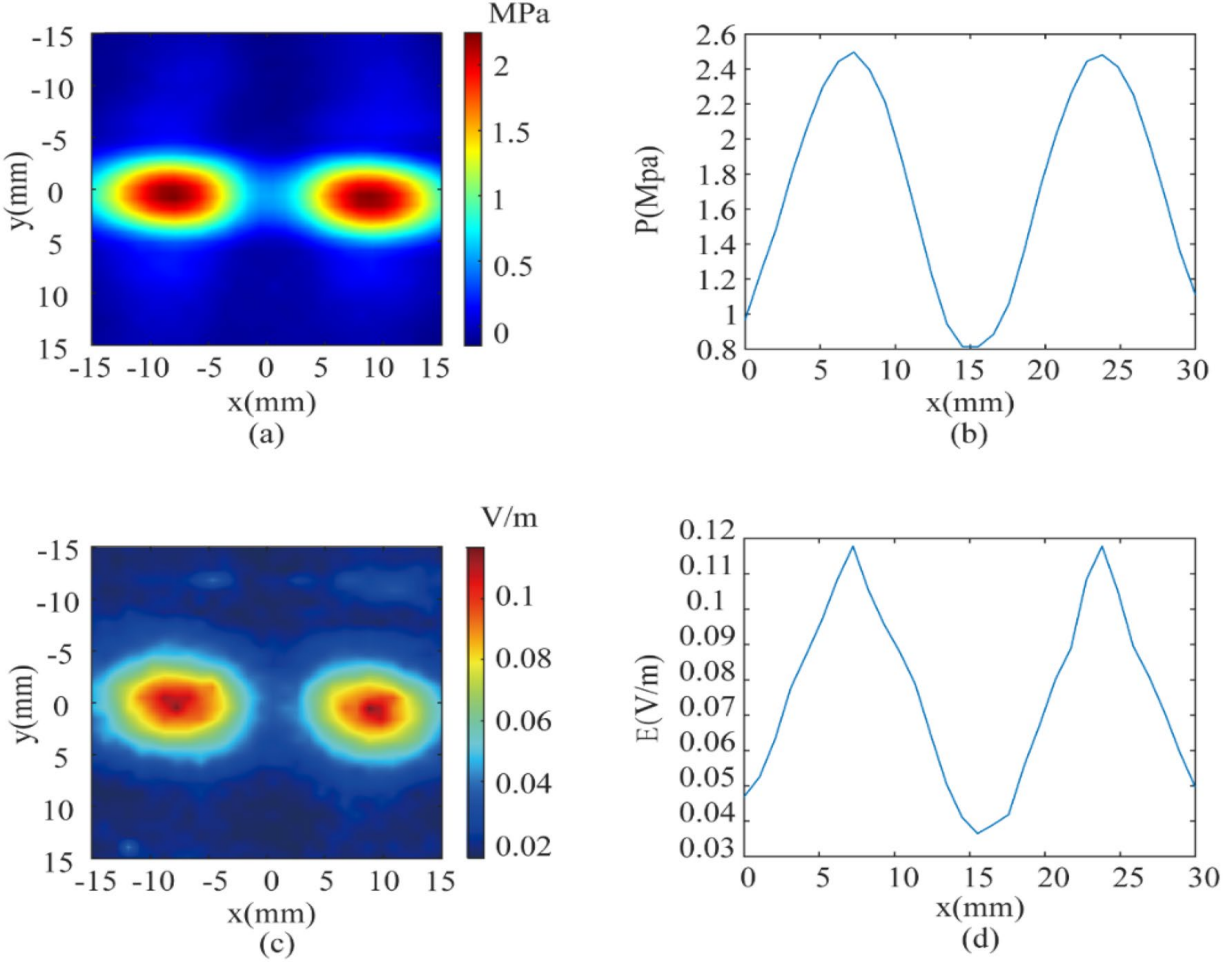
Distribution of acoustic pressure field and electric field intensity at the measured 500 kHz dual-target. (**a**) xy-plane acoustic pressure field, (**b**) distribution of the acoustic pressure field along the x-axis of y = 0, (**c**) xy-plane electric field intensity and (**d**) distribution of the electric field intensity along the x-axis at y = 0.

The ultrasound and magnetic field parameters mentioned above are used in experimental measurements. The two focal points are set at (− 9, 0, − 50) and (9, 0, − 50). At the distance from the transducer 50 mm, the acoustic pressure and electric field intensity distributions are measured and compared by moving the hydrophone and self-made electrodes with 1 mm steps in the x and y directions, respectively. Figure 8a and Fig. 8b show the distribution of the acoustic pressure in the xy-plane and on the x-axis at y = 0, respectively. It can be seen that the two focal points are symmetrical about the central axis of the x = 0. The actual distance between the two focuses is 16 mm and the maximum acoustic pressure of both focal points is 2.5 MPa. In Fig. 8a, the long axis focusing length of the left target is 7.8 mm and the short axis focusing length is 4.2 mm. The long axis focusing length of the right target is 6.5 mm, and the short axis focusing length is 4.1 mm. The total focusing area of the two targets is 49.1 mm^2^, which shows that the acoustic pressure field distribution of the two targets has a high degree of consistency. Figure 8c,d show the electric field intensity distribution of the xy-plane and the x-axis at y = 0, respectively. It can be seen that the electric field distributions of the two targets are basically the same, and the maximum voltage signals are 0.11 mV and 0.105 mV, respectively. Based on the distance between the plates, the electric field intensity at the dual-target electric stimulation can be calculated to be 0.11 V/m and 0.105 V/m, respectively, and the total focusing area at the two focal points is 39.7 mm^2^. In Fig. 8c, the long axis focusing length of the left target is 5.7 mm and the short axis focusing length is 4.9 mm. The long axis focusing length of the right target is 4.5 mm and the short axis focusing length is 3.9 mm. The actual electric field intensity is less than the theoretical value in relative simulation, because the voltage signal intensity measured by the self-made electrode is low, vulnerable to noise interference, and there is impedance between the two metal plates. It can be seen from Fig. 8 that the actual distribution of the acoustic pressure field and the electric field intensity is indeed highly consistent, and the focusing area of the electric field intensity is slightly smaller than the focusing area of the acoustic pressure field. Therefore, dual-target magneto-acoustic coupling electrical stimulation with spatial resolution of mm-level at the deep brain can be realized by using the high focus of ultrasound and phased-array focusing technology.

**Figure 9:**
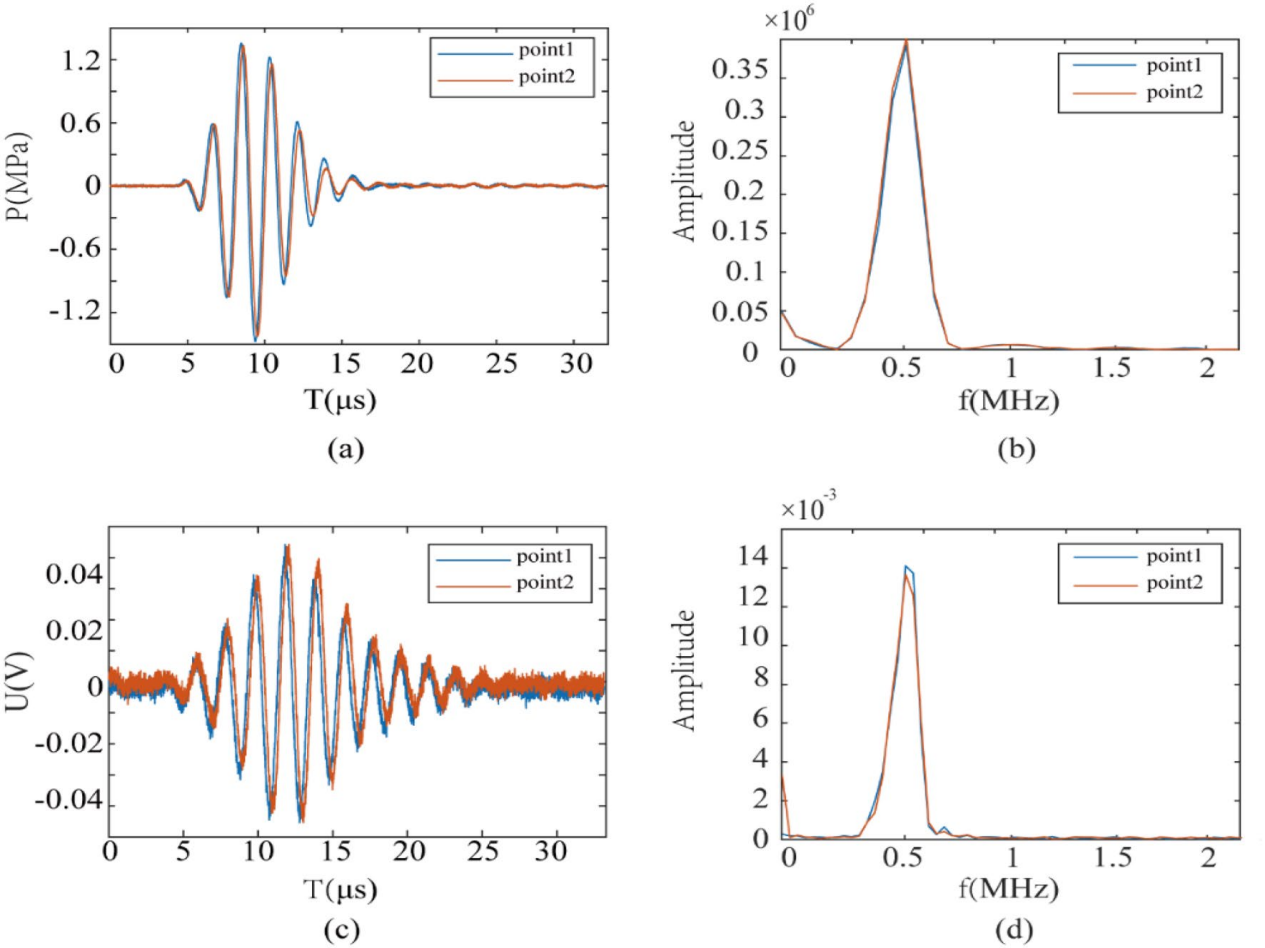
Ultrasonic and voltage signals at the dual target sites. (**a**) Time-domain and (**b**) amplitude-frequency response of ultrasound signal received by the hydrophone; (**c**) time-domain electrical signal and (**d**) amplitude-frequency response received by the electrodes.

To further analyze the acoustic signals and electrical signals at the targets, Fig. 9 show the comparison of acoustic signals and voltage signals at the two target points in time domain and frequency domain. From the comparison of Fig. 9a–d, we can gain that the signals at the two targets, whether acoustic or electrical, are consistent in the time domain and frequency domain. It shows that the two target signals are consistent and independent of each other without interfering with each other. The time domain and frequency domain of the electrical signal are consistent with the acoustic signal and the electrical stimulation is indeed generated by focused ultrasound, which verifies the magneto-acoustic coupling theory. The experimental results further confirmed the feasibility of the in experimental measurements. Multi-target TMAES, and the main frequency and focusing characteristics of the excitation ultrasound, as well as the corresponding focusing mode, can be adjusted according to the actual multi-target electrical stimulation needs.

**Figure 10:**
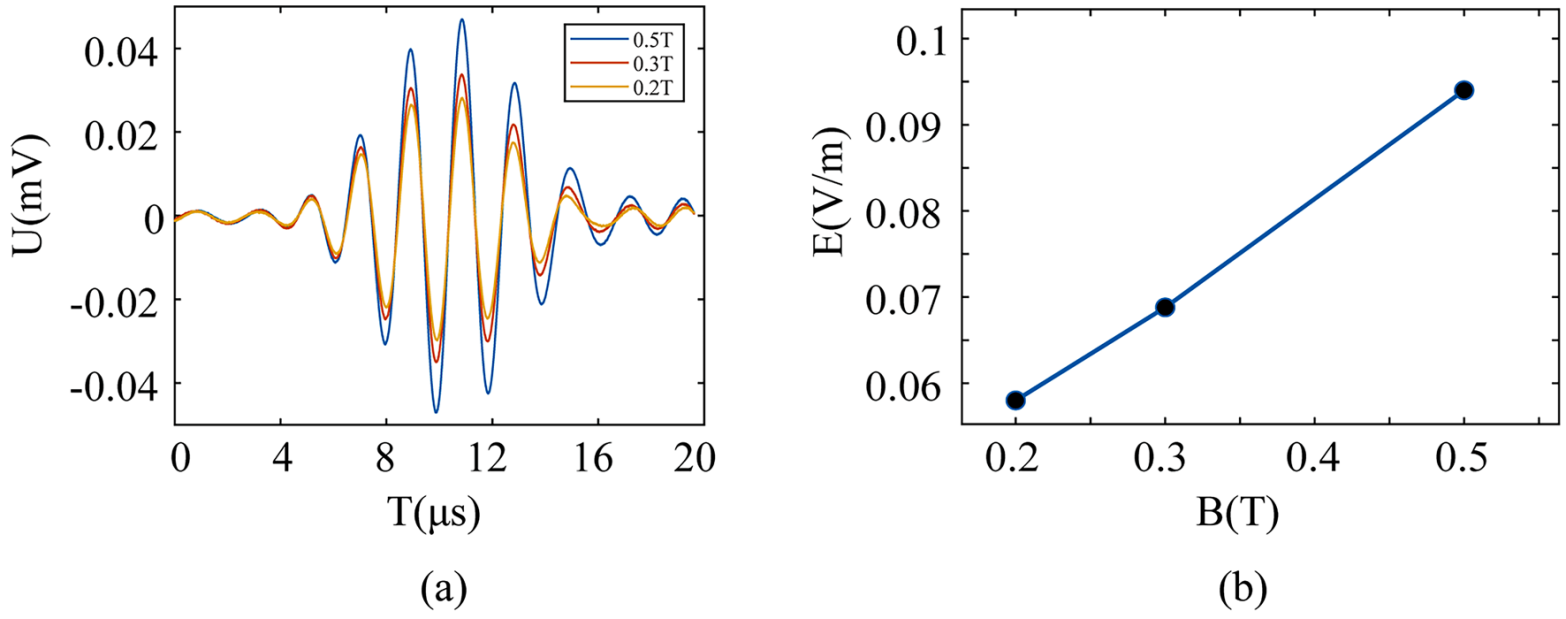
(**a**) Electrical signal waveforms and (**b**) peak-to-peak values at different magnetic induction intensities.

Furthermore, we studied the relationship between the magnetic induction intensity and magneto-acoustic coupling electrical signals in saline. This can provide a new strategy for improving the intensity of electrical stimulation in multi-targets TMAES. The magnetic induction intensity ***B*** in the target area is adjusted by changing the distance between the magnet and the target area. The distance we are talking about is the straight line distance between the magnet surface and the set target point. We use a magnet with a surface induction of 0.5 T, ***B*** = 0.3 T at 10 mm from the magnet and ***B*** = 0.2 T at 15 mm from the magnet. The magnetic induction strength was detected by a Gauss meter (Lakeshore460, LakeShore, Amercia). Keeping the ultrasound excitation with *f* = 500 kHz at ***B*** = 0.2 T, ***B*** = 0.3 T and ***B*** = 0.5 T, the corresponding stimulus electrical signals are shown in Fig. 10. We find that the amplitude of the magneto-acoustic coupled electrical signal is positively correlated with the magnetic induction intensity. In multi-target TMAES technology, the electrical stimulation intensity can be improved by increasing the magnetic induction intensity to reach the ideal stimulation threshold under the same conditions of ultrasound excitation.

## Discussion

Based on Eq. (9) and Figs. 5 and 6, the distribution of the electric field can be approximately equal to the distribution of the acoustic field. It is known that the frequency of ultrasound affects the acoustic penetration depth and the size of the acoustic focusing target point, which is closely related to the distribution of the acoustic field. Therefore, the frequency of ultrasound also determines the distribution of the magneto-acoustic electric field.

**Figure 11:**
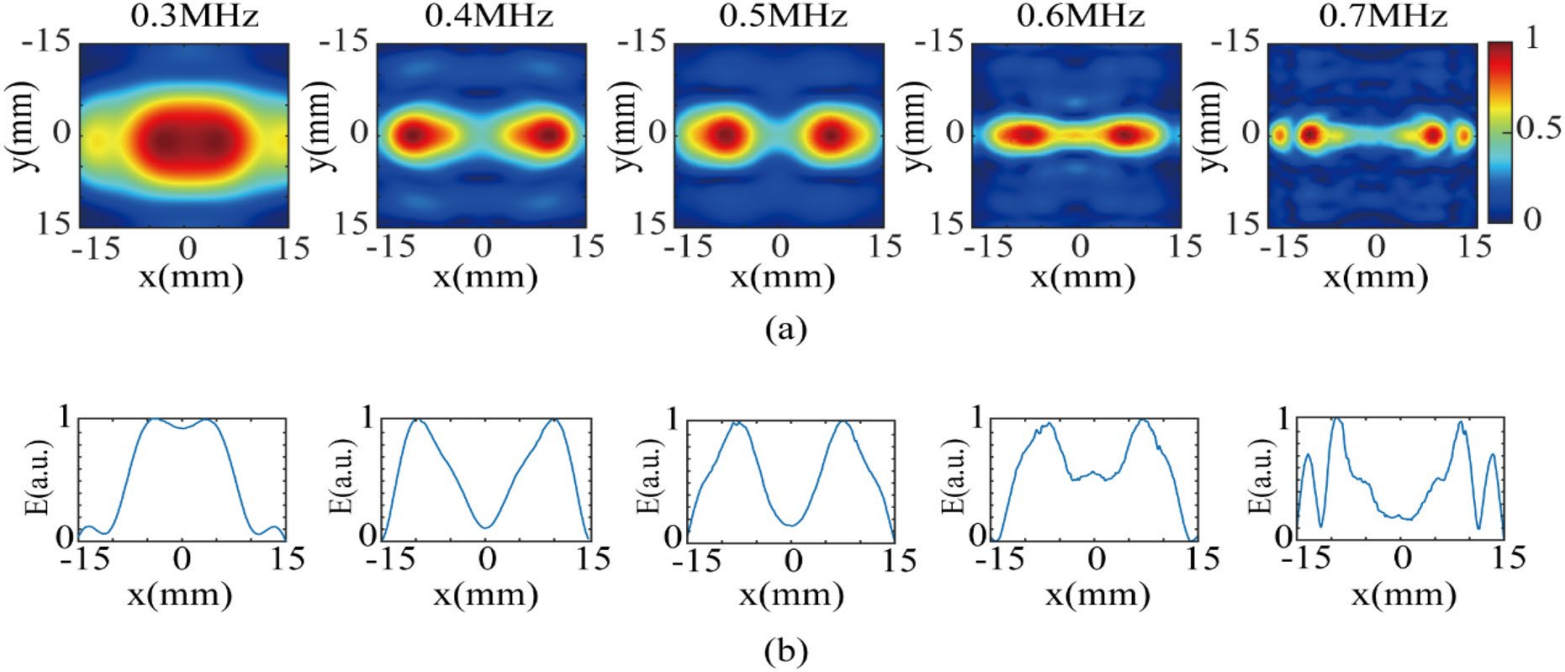
Normalised distribution of the electric field intensity at different frequencies. (**a**) Electric field intensity distribution in the xy plane at z = 50 mm. (**b**) Corresponding X profiles of the electric field intensity distribution at y = 0.

In order to study the effect of ultrasonic frequency on the distribution of dual-target magneto-acoustic electric field, the ultrasonic transducer was made to emit focused acoustic of 300 kHz, 400 kHz, 500 kHz, 600 kHz and 700 kHz. The normalized electric field distribution of the xy-plane at z = 50 mm with different frequencies is obtained, as shown in Fig. 11a. The corresponding x-axis distribution of the electric field intensity at y = 0 is drawn, as shown in Fig. 11b.

According to the definition of the focusing area above, the total focusing areas at different frequencies are calculated to be 200 mm^2^, 69 mm^2^, 65 mm^2^, 39 mm^2^, and 35.8 mm^2^. We specify that the range length of the maximum attenuation of 3 dB of the electric field intensity on the x-axis is determined to be the focusing length. To quantify the separation degree of the two focus targets, this paper introduces an evaluation index $R = \frac{{\left[ {E_{\max } - E_{(x = 0mm)} } \right]}}{{\left[ {E_{\max } - E_{\min } } \right]}}$, where $E_{\max }$ and $E_{\min }$ are the maximum and minimum values of the electric field, respectively. The value of R is used to evaluate the separation degree of the two targets. The higher the value is, the greater the separation degree is. R = 1 indicates that the two targets are completely separated. The results of the evaluation are summarized in Table 2.

**Table 2:** Evaluation results of magneto-acoustic coupling electrical stimulation distribution of the two targets at different frequencies.

Frequency (kHz)	X-axis focusing length (mm)		Distance between two targets (mm)	Total focusing area (mm^2^)	R
	Left target	Right target			
300	9.5	9.3	7.5	200	0.0801
400	8.2	8.0	19.4	69	0.8927
500	7.1	7.0	15.2	65	0.8581
600	7.1	7.1	13.7	39	0.4426
700	4.2	4.2	17.9	35.8	0.8105

As seen from Fig. 11 and Table 1, at the frequency of 300 kHz, the focusing areas of the two targets have overlapping parts, and so dual-target electrical stimulation cannot be achieved. When the frequency is 700 kHz, the side lobe of ultrasound is more obvious, which affects the focusing effect. In the range of 400–600 kHz, the area of the focusing area decreases with increasing frequency. Therefore, in the design of multi-target electrical stimulation, in addition to the stimulation depth, the effects of ultrasound frequency on focal area need to be considered comprehensively.

This research focused on a new brain stimulation method: multi-target TMAES. And the feasibility of this method was confirmed through software simulation and experimental verification. The findings from this study lay the foundation for further exploration and optimization of these novel therapeutic approaches in the field of brain functional disorders.

Based on Figs. 7 and 11, the main frequency and focus characteristics of the excitation ultrasound, as well as the corresponding focus mode, can be adjusted according to the actual multi-target electrical stimulation needs. In multi-target TMAES technology, the electrical stimulation intensity can be improved by increasing the magnetic induction intensity to reach the ideal stimulation threshold under the same conditions of ultrasound excitation in Figs. 6 and 10.

Moreover, different directions of nerve currents affect nerve stimulation results^31^. There are also differences in the direction in which different subdivisions of the human brain are stimulated. Based the formula of magnetic-acoustic coupling theory, as shown in Eqs. (8) and (9) of the manuscript, the direction of the electric field strength is related to the direction of the magnetic induction strength. We can change the direction of the magnetic field so that multiple targets are electrically stimulated from different directions at the same time.

**Figure 12:**
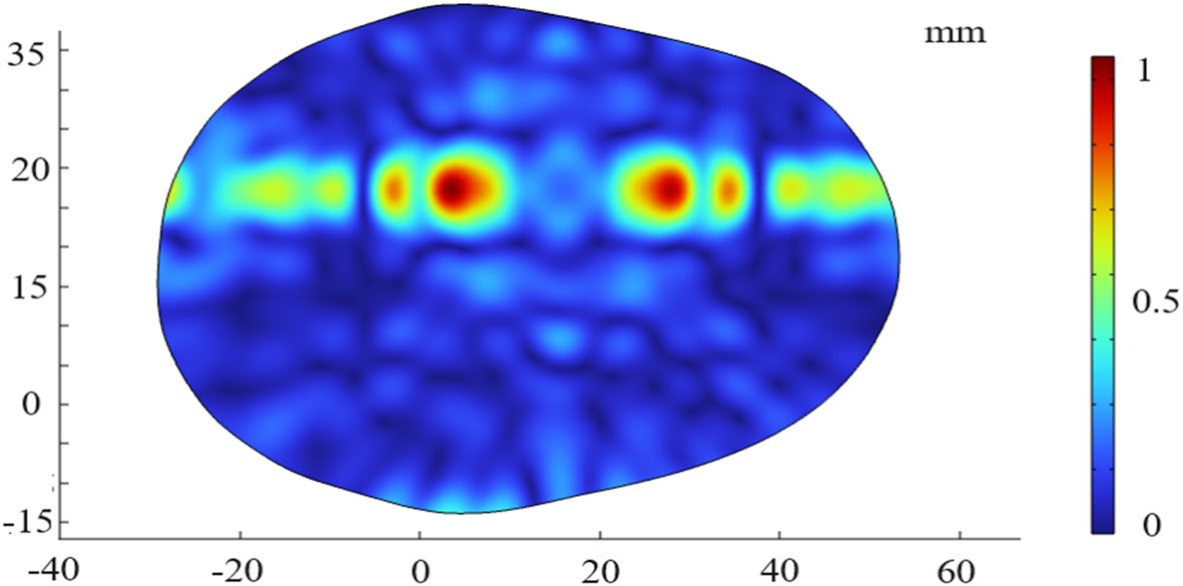
Simulation result of single-layer cranium electric field under double target.

However, it is confirmed only in saline, and the possible effects of phase distortion, energy attenuation, and reflection and refraction of ultrasound due to cranial bones, scalp, etc., are not taken into account. Therefore, in the simulation software, we simulate the electric field distribution of two targets under the real skull model of a single layer. In order to reduce the calculation amount of simulation, the skull model is selected to be scaled down in equal proportions, and the simulation results are shown as follows. The next step is to improve the ultrasonic focusing algorithm to consider the influence of multi-layer brain tissue and improve the precision of multi-target stimulus focusing. Furthermore, the transducer structure, ultrasonic frequency and focusing law were found to have a large influence on the focused acoustic pressure and electric field intensity distribution of multi-target. Taking into account the different distances between different subdivisions of the human brain during the actual stimulation, more attempts can be made on the structure of the transducer, frequency and multi-target focusing law when applying in practice according to the stimulation demand. Combined with the improved focusing algorithm, the most suitable multi-target stimulation scheme can be found to achieve high-resolution noninvasive multi-target electrical stimulation in a magnetic field environment (Fig. 12).

## Conclusion

In this paper, a novel multi-target TMAES method is proposed by combining the magneto-acoustic coupling theory with phased array ultrasound focusing technology. The proposed method can achieve noninvasive multi-target high spatial resolution electrical stimulation at the mm-level. A simulation model and an experimental system of multi-target TMAES are established. Both the simulation results and experimental process proved the effectiveness of the method. Under the same other conditions, the intensity of the stimulated electric field decreases with the increase in the number of target points. Based on the magneto-acoustic coupling formula, the intensity of the electric field can also increase by increasing the magnetic induction intensity. In addition, we study the multi-target electrical stimulation with different stimulation intensities and discuss the relationship between ultrasound frequency and the distribution of stimulated electric field.

Based on phased array focusing technology and magneto-acoustic coupling theory, this method can quickly and flexibly tune the targets position, stimulation parameters, as well as the magnetic field environment, so as to achieve focused electrical stimulation at different deep brain region. This proposed multi-target TMAES method provides a novel tool for exploring brain functional connectivity, the pathogenesis of brain diseases, and the diagnosis and rehabilitation treatment of major neuropsychiatric disorders. In the future, the multi-target TMAES technique would be applied to the studies of the brain diseases and brain network research using animals.

## Data Availability

Data will be available upon request from Zhipeng Liu.

## References

[1] Raichle, M. E. The brain’s default mode network. *Annu. Rev. Neurosci.* **38**, 433–447 (2015). 25938726 10.1146/annurev-neuro-071013-014030

[2] Thiebaut, D. S. M. & Forkel, S. J. The emergent properties of the connected brain. *Science.* **378**, 505–510 (2022). 36378968 10.1126/science.abq2591

[3] Mueller, K. *et al.* Brain connectivity changes when comparing effects of subthalamic deep brain stimulation with levodopa treatment in Parkinson’s disease. *NeuroImage Clin.* **19**, 1025–1035 (2018). 30035027 10.1016/j.nicl.2018.05.006 PMC6051673

[4] Johnson, L. A. *et al.* Direct activation of primary motor cortex during subthalamic but not pallidal deep brain stimulation. *J. Neurosci.* **40**, 2166–2177 (2020). 32019827 10.1523/JNEUROSCI.2480-19.2020 PMC7055133

[5] Cao, J. *et al.* Brain functional and effective connectivity based on electroencephalography recordings: A review. *Hum. Brain Mapp.* **43**, 860–879 (2022). 34668603 10.1002/hbm.25683 PMC8720201

[6] Lizarraga, K. J., Jagid, J. R. & Luca, C. C. Comparative effects of unilateral and bilateral subthalamic nucleus deep brain stimulation on gait kinematics in Parkinson’s disease: A randomized, blinded study. *J. Neurol.* **263**, 1652–1656 (2016). 27278062 10.1007/s00415-016-8191-3

[7] Weise, D. *et al.* Unilateral multi-target deep brain stimulation in hemidystonia and hemichoreoathetosis following ischemic thalamic stroke. *Basal Ganglia.* **6**, 153–156 (2016).

[8] Lehner, A. *et al.* Multisite Rtms for the treatment of chronic tinnitus: Stimulation of the cortical tinnitus network—A Pilot Study. *Brain Topogr.* **26**, 501–510 (2013). 23229756 10.1007/s10548-012-0268-4

[9] Kleinjung, T. *et al.* Combined temporal and prefrontal transcranial magnetic stimulation for tinnitus treatment: A pilot study. *Otolaryngol. Head Neck Surg.* **138**, 497–501 (2008). 18359361 10.1016/j.otohns.2007.12.022

[10] Zhang, X. *et al.* Multi-target and multi-session transcranial direct current stimulation in patients with prolonged disorders of consciousness: A controlled study. *Front. Neurosci.* **15**, 641951 (2021). 34566555 10.3389/fnins.2021.641951 PMC8456025

[11] Saturnino, G. B., Madsen, K. H., Siebner, H. R. & Thielscher, A. How to target inter-regional phase synchronization with dual-site transcranial alternating current stimulation. *Neuroimage.* **163**, 68–80 (2017). 28919407 10.1016/j.neuroimage.2017.09.024

[12] Miyaguchi, S. *et al.* Gamma tacs over M1 and cerebellar hemisphere improves motor performance in a phase-specific manner. *Neurosci. Lett.* **694**, 64–68 (2019). 30445151 10.1016/j.neulet.2018.11.015

[13] Memarian, S. M. & Denison, T. A neurostimulator system for real, sham, and multi-target transcranial magnetic stimulation. *J. Neural Eng.* **19**, 026035 (2022). 10.1088/1741-2552/ac60c9 PMC7614713 35325879

[14] Pacheco-Barrios, K. *et al.* Methods and strategies of Tdcs for the treatment of pain: Current status and future directions. *Expert Rev. Med. Devices.* **17**, 879–898 (2020). 32845195 10.1080/17434440.2020.1816168 PMC7674241

[15] Woods, A. J. *et al.* A technical guide to Tdcs, and Related non-invasive brain stimulation tools. *Clin. Neurophysiol.* **127**, 1031–1048 (2016). 26652115 10.1016/j.clinph.2015.11.012 PMC4747791

[16] Klírová, M. et al. Modulating inhibitory control processes using individualized high definition theta transcranial alternating current stimulation (Hd Θ-Tacs) of the anterior cingulate and medial prefrontal cortex. *Front. Syst. Neurosci*. **15**, (2021). 10.3389/fnsys.2021.611507 PMC8042221 33859554

[17] Klomjai, W., Katz, R. & Lackmy-Vallée, A. Basic principles of transcranial magnetic stimulation (Tms) and repetitive Tms (Rtms). *Ann. Phys. Rehabil. Med.* **58**, 208–213 (2015). 26319963 10.1016/j.rehab.2015.05.005

[18] Zhu, Z. & Yin, L. A mini-review: Recent advancements in temporal interference stimulation in modulating brain function and behavior. *Front. Hum. Neurosci.* **17**, (2023). 10.3389/fnhum.2023.1266753 PMC10539552 37780965

[19] Beisteiner, R., Hallett, M. & Lozano, A. M. Ultrasound neuromodulation as a new brain therapy. *Adv. Sci.* **10**, 2205634 (2023). 10.1002/advs.202205634 PMC10190662 36961104

[20] Grossman, N., Okun, M. S. & Boyden, E. S. Translating temporal interference brain stimulation to treat neurological and psychiatric conditions. *JAMA Neurol.* **75**, 1307–1308 (2018). 30264149 10.1001/jamaneurol.2018.2760

[21] Darmani, G. *et al.* Non-invasive transcranial ultrasound stimulation for neuromodulation. *Clin. Neurophysiol.* **135**, 51–73 (2022). 35033772 10.1016/j.clinph.2021.12.010

[22] Zhuang, X. *et al.* A spatial multitarget ultrasound neuromodulation system using high-powered 2-D array transducer. *IEEE Trans. Ultrason. Ferroelectr. Freq. Control.* **69**, 998–1007 (2022). 34990356 10.1109/TUFFC.2022.3140889

[23] He, J. *et al.* Multitarget transcranial ultrasound therapy in small animals based on phase-only acoustic holographic lens. *IEEE Trans. Ultrason. Ferroelectr. Freq. Control.* **69**, 662–671 (2022). 34847028 10.1109/TUFFC.2021.3131752

[24] He, J. *et al.* Simultaneous multi-target ultrasound neuromodulation in freely-moving mice based on a single-element ultrasound transducer. *J. Neural Eng.* **20**, 16021 (2023). 10.1088/1741-2552/acb104 36608340

[25] Chu, F. *et al.* Transcranial magneto-acoustic stimulation attenuates synaptic plasticity impairment through the activation of Piezo1 in Alzheimer’s disease mouse model. *Research.* **6**, 0130 (2023). 37223482 10.34133/research.0130 PMC10202414

[26] Verhagen, L. *et al.* Offline impact of transcranial focused ultrasound on cortical activation in primates. *Elife.* **8**, e40541 (2019). 30747105 10.7554/eLife.40541 PMC6372282

[27] Norton, S. J. Can ultrasound be used to stimulate nerve tissue?. *Biomed. Eng. Online.* **2**, 6 (2003). 12702213 10.1186/1475-925X-2-6 PMC153496

[28] Wang, H. *et al.* Comparative study of transcranial magneto-acoustic stimulation and transcranial ultrasound stimulation of motor cortex. *Front. Behav. Neurosci.* **13**, 241 (2019). 31680896 10.3389/fnbeh.2019.00241 PMC6798265

[29] Zhao, S., Liu, D., Liu, M., Luo, X. & Yuan, Y. Theoretical Analysis of Effects of Transcranial Magneto-Acoustical Stimulation On Neuronal Spike-Frequency Adaptation. *Bmc Neurosci.* **23**, 26 (2022). 35501687 10.1186/s12868-022-00709-9 PMC9063290

[30] Zhang, S. *et al.* Effect of transcranial ultrasonic-magnetic stimulation on two types of neural firing behaviors in modified Izhikevich model. *IEEE Trans. Magn.* **54**, 1–4 (2018).

[31] Montalibet, A., Jossinet, J., Matias, A. & Cathignol, D. Electric current generated by ultrasonically induced lorentz force in biological media. *Med. Biol. Eng. Comput.* **39**, 15–20 (2001). 11214267 10.1007/BF02345261

